# The aspects of microbial biomass use in the utilization of selected waste from the agro-food industry

**DOI:** 10.1515/biol-2020-0099

**Published:** 2020-10-22

**Authors:** Marek Kieliszek, Kamil Piwowarek, Anna M. Kot, Katarzyna Pobiega

**Affiliations:** Department of Food Biotechnology and Microbiology, Institute of Food Sciences, Warsaw University of Life Sciences—SGGW, Nowoursynowska 159C, 02-776 Warsaw, Poland

**Keywords:** biomass, agriculture waste, metabolites, industrial applications, microorganisms

## Abstract

Cellular biomass of microorganisms can be effectively used in the treatment of waste from various branches of the agro-food industry. Urbanization processes and economic development, which have been intensifying in recent decades, lead to the degradation of the natural environment. In the first half of the 20th century, problems related to waste management were not as serious and challenging as they are today. The present situation forces the use of modern technologies and the creation of innovative solutions for environmental protection. Waste of industrial origin are difficult to recycle and require a high financial outlay, while the organic waste of animal and plant origins, such as potato wastewater, whey, lignin, and cellulose, is dominant. In this article, we describe the possibilities of using microorganisms for the utilization of various waste products. A solution to reduce the costs of waste disposal is the use of yeast biomass. Management of waste products using yeast biomass has made it possible to generate new metabolites, such as β-glucans, vitamins, carotenoids, and enzymes, which have a wide range of industrial applications. Exploration and discovery of new areas of applications of yeast, fungal, and bacteria cells can lead to an increase in their effective use in many fields of biotechnology.

## 1 Introduction

Because of the depletion of natural resources due to increasing technological development, determined and coherent action is required to treat waste as a valuable resource that can be reused or recycled. The continuous economic development also contributes to an increase in the accumulation of production waste. Therefore, measures should be taken to manage waste economically and, above all, to protect the natural environment. These measures might help to preserve natural resources and improve environmental balance. Special attention has been paid to the use of microorganisms in biotechnological research. Among many examples, yeasts are widely known and used in many branches of the agro-food industry. One of the characteristic functions of yeasts is waste biodegradation. The usefulness of these microorganisms lies in the possibility of utilizing their metabolic products in food technology and protection of the environment from industrial waste at reduced economic costs. Thanks to the progress in technology and the development of research methods, new strains with unique characteristics and abilities are constantly being discovered. Such microorganisms have the ability to transform raw materials rich in proteins, carbohydrates, and fats. The search for innovative technologies for the use of yeast strains might result in the continuous advances in biotechnology, which in turn can enable the reduction and management of industrial waste. An important objective of waste utilization is to obtain full-value products from yeast cells, which can be exemplified by biomass, protein hydrolysates, and B vitamins. By using modern methods of molecular biology to modify yeast cells, it is possible to multiply the synthesis of these compounds. In this way, organisms with enhanced production characteristics can be obtained. However, it should be noted that the use of strains modified by biotechnological processes may lead to various adverse effects. This is due to the fact that it is impossible to predict the effects that may be caused by the modified microorganisms. Another solution to improve the economic aspect of metabolite production using yeast is the preparation of microbiological media from cheap waste materials that can support the biosynthesis of individual metabolites under appropriate culture conditions. A reason for the continuous research conducted in the direction of pro-ecological applications of yeast is the increase in contamination due to the surplus production of raw materials subject to decomposition. Waste left unprocessed may trigger the growth of pathogenic microorganisms, leading to environmental degradation and the development of various diseases. The examples presented in this article may provide opportunities for the development of innovative concepts of using microorganisms in waste management with the simultaneous possibility of obtaining attractive products.

In this article, different methods of waste disposal using microorganisms are discussed. Microorganisms allow for the transformation of postproduction components into valuable raw materials. The methods of utilization of yeast cells include, among others, fermentation processes and biosynthesis of vitamins, enzymes, and biosurfactants.

## 2 Selected waste from the agro-food industry

### 2.1 Potato wastewater

**Figure 1:**
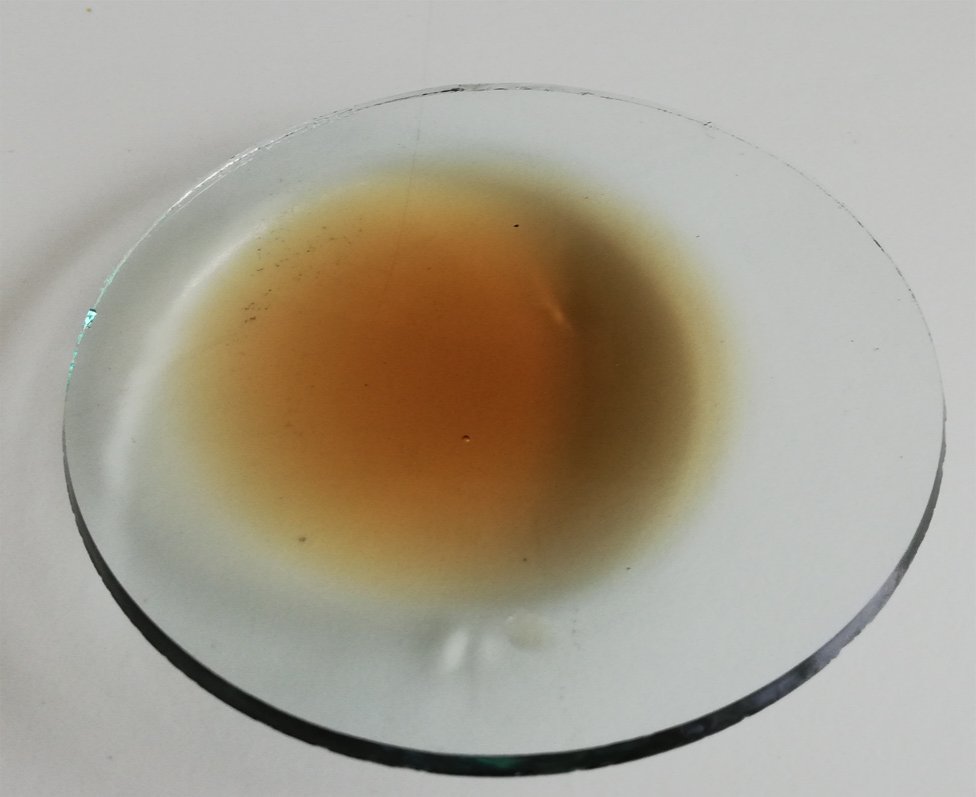
Potato wastewater.

The food industry generates a significant amount of wastewater rich in biogenic elements and organic compounds. Their utilization is troublesome and burdened with high costs. Some waste from potato processing, including potato wastewater formed during the production of starch, can be used as a source of nitrogen and mineral components for yeast. It is estimated that processing of 1,000 Mg (megagram) of potatoes results in the production of 600 m^3^ of potato wastewater [1]. Restrictions imposed by the European Union on the discharge of industrial wastewater into the environment forced the development of new solutions for the utilization of wastes. One of these is to use the liquid waste from the food industry as a cheap microbiological medium. Potato wastewater (Figure 1) contains both inorganic (1%) and organic substances (4%) and is a rich source of proteins and vitamins, mainly the B group [2,3].

It also contains substances that affect the absorption of minerals such as the salts of phytic acid and toxic glycoalkaloids [4,5]. Proteins contained in the juice are considered to be the main factor behind the nutritional and functional value of potato wastewater. Their amino acid composition resembles that of egg protein [6]. To reduce the burden of potato wastewater, a process called acid–thermal coagulation is carried out to remove nitrogen compounds. However, even after the so-called deproteination process, the chemical composition of potato wastewater is characterized by the richness of various nutrients. The release of large quantities of waste from the potato-processing industry is a serious environmental problem due to the high chemical and biochemical oxygen demand (COD and BOD5, respectively). Therefore, new environmental-friendly methods for the utilization of potato wastewater are being sought [2].

An interesting application of potato wastewater is as a culturing medium for the growth of microorganisms in biotechnological processes. One example is the use of potato wastewater in the production of lipids of microbiological origin. A study conducted by Muniraj et al. [7] confirmed the possibility of using potato wastewater in the production of lipids with various strains of *Aspergillus oryzae*. The obtained results indicated the ability of *A. oryzae* mold to assimilate compounds contained in potato wastewater and biosynthesize single-cell oil with a high fat content (2.8 g/L). Abanoz et al. [8] used potato wastewater as a substrate for ethanol production with recombinant *Escherichia coli* strains. Potato wastewater can also be utilized as a substrate for the biosynthesis of lactic acid, which is used on a large scale in the food and fermentation industry, using molds of *Rhizopus arrhizus*.

In a study conducted by Nowak et al. [9], *Aspergillus niger* NRRL 334 was successfully cultivated in a medium with deproteinized potato wastewater, achieving a biomass yield of 13 g/L of sewage and simultaneous reduction of COD index by 58%. Huang et al. [10] demonstrated that *R. arrhizus* DAR 36017 cultured with 10 mg of CaCO_3_/L produced 21 g of lactic acid/L of culture due to complete saccharification of starch contained in the substrate within 28 h. Liu et al. [11] designed a single-cell protein (SCP) preparation from a mixed culture of *Bacillus pumilus*, *Candida utilis*, and *A. niger* cells at a ratio of 7:2:1, which contained about 46% of crude protein. Muniraj et al. [12] successfully produced fat and γ-linolenic acid using two fungal species, *Aspergillus flavus* and *Mucor rouxii*. In addition, they noted a decrease in the value of BOD5 and COD indices as well as in the content of phosphorus and nitrogen in the substrate by over 50%.

The studies carried out by Kieliszek et al. [13] showed that potato wastewater can be used as a medium for growing *C. utilis* ATCC 9950 yeast. This yeast cell biomass can be used in the production of vitamin–mineral preparations and supplements. Feed yeast not only is a valuable source of many vitamins, enzymes, and proteins but also plays an important role as a carrier of essential bioelements (e.g., selenium). Bzducha-Wróbel et al. [14,15] used potato wastewater as a base for the biosynthesis of (1,3)/(1,6)-glucans. These polysaccharides are characterized by many antimutagenic and antioxidant properties and reduce the plasma level of low-density lipoprotein cholesterol. Due to these effects, they are used, *inter alia*, in the pharmaceutical industry.

Thus, the use of waste materials from the potato-processing industry in the biosynthesis of products such as ethanol and lactic acid not only contributes to the reduction of production costs but also has a great significance in the reduction of environmental pollution.

### 2.2 Glycerol

**Figure 2:**
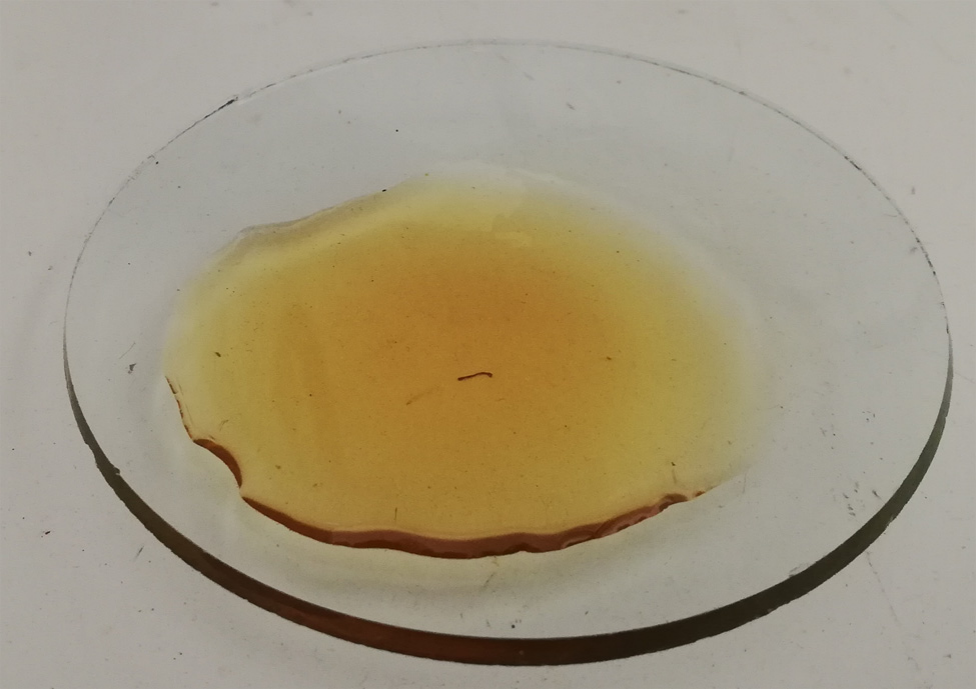
Glycerol.

In recent years, new waste materials that can be used in biotechnological processes have been sought, concurrently making it possible to recycle them economically. One such raw material is glycerol (Figure 2), which is a by-product resulting from the production of fatty acid methyl esters (biodiesel). During the production process, glycerin (polar) and ester (nonpolar) phases are formed [16].

The glycerin phase consists of approximately 40–70% of glycerol; the remaining part consists of impurities such as methanol, soaps, and trace amounts of heavy metals [17]. Glycerin is subjected to a multistage purification process to obtain glycerol, which can be used in the pharmaceutical, cosmetic, food, and chemical industries. In order to be used in industries, glycerol must be subjected to a purification process involving deodorization, bleaching, and ion exchange. This is an expensive process, requiring high financial outlays that small enterprises cannot afford [18]. Therefore, much cheaper methods of biodegradation are used.

The growing demand for fossil fuels and the simultaneous depletion of their resources are the reasons for the search for alternative sources of energy. Currently, the idea of producing fuels from organic raw materials, including oilseeds, is of great interest to entrepreneurs [17]. Biodiesel is a fuel that can be easily used in conventional engines. Its popularization is undoubtedly hindered by high production costs, primarily as a result of the need to utilize the resulting waste glycerol. A solution for the management of waste glycerol is to use it as a component of culture media for microorganisms [19,20]. It was found that glycerol can be assimilated by yeast cells by means of facilitated diffusion and then used as a source of energy for their metabolic changes [21,22]. Using certain bacteria, glycerol can be converted by biotransformation to 1,3-propanediol, which is a desirable raw material in various industries including chemical and automotive [23]. A study performed by Santos et al. [24] showed that the yield of yeast biomass from *Yarrowia* and *Candida* spp. was high when the only carbon source in the medium was glycerol. *Yarrowia lipolytica* also has the ability to produce citric acid in the medium containing glycerol [23]. Choi et al. [25] demonstrated that the rate of synthesis of ethanol by *Kluyvera cryocrescens* S26 bacteria was 94% when the only carbon source in the medium was glycerol. The best known and widely used biopolymer synthesized by microorganisms is polyhydroxybutyrate. It has properties similar to those of polypropylene, but has a clear advantage because it is not toxic to humans. This favors the use of polyhydroxybutyrate in the production of bone implants or as a scaffold in the production of human tissues [26]. Mothes et al. [27] demonstrated that *Cupriavidus necator* and *Paracoccus denitrificans* were able to efficiently synthesize this polymer in a medium containing waste glycerol. Another example of substances produced by microorganisms is biosurfactants. These compounds show amphiphilic properties (as they have lipophilic and hydrophilic fragments) and are classified into several different groups (e.g., glycolipids or lipopeptides). Due to their different molecular structures and low toxicity, as well as easy biodegradability, they are used in many branches of industry (e.g., cosmetic, pharmaceutical, food, and chemical) [28]. Liu et al. [29] demonstrated that the fungus *Ustilago maydis* parasitizing on maize cobs has the ability to bioconvert waste glycerol to glycolipid-type biosurfactants.

Thus, waste glycerol is a good culturing medium for various microorganisms. Its use in biotechnology can reduce the cost of producing large quantities of substances such as citric acid. However, new ideas for biotechnological waste treatment need to be developed, as biodiesel production is expected to increase further and, as a result, huge amounts of waste which is difficult to recycle are expected to be generated.

### 2.3 Apple pomace

**Figure 3:**
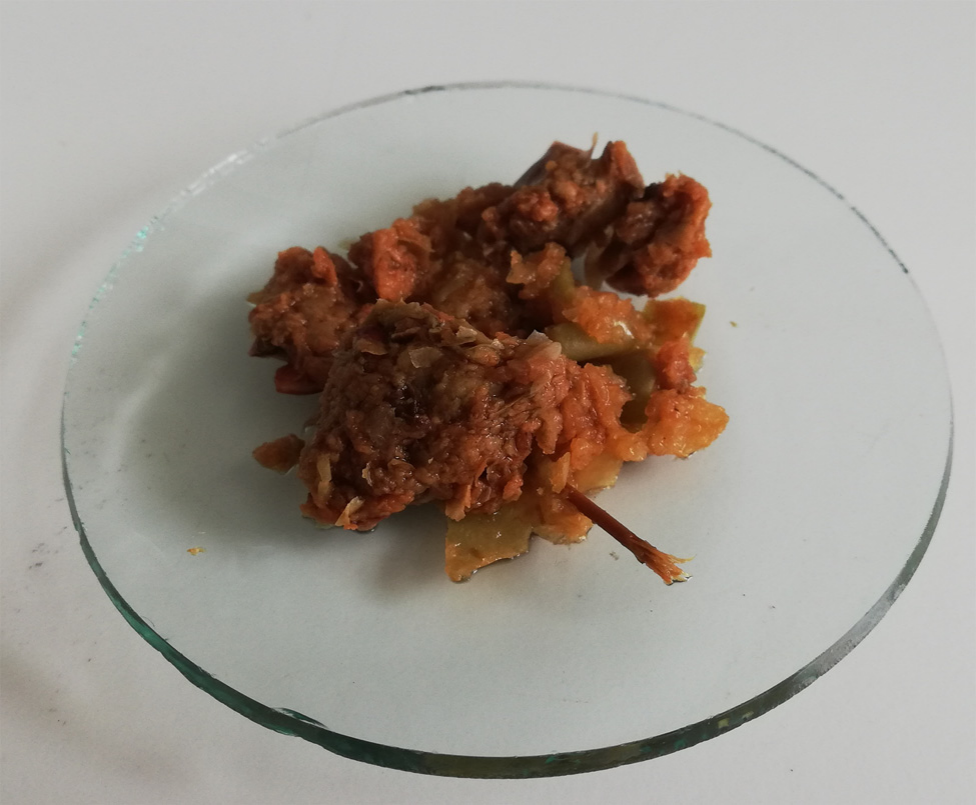
Apple pomace.

Fruit processing results in the accumulation of waste and wastewater. The vast majority of apples destined for processing are pressed to produce juices or alcoholic beverages, generating a significant amount of peel and seed waste and pulp, collectively referred to as apple pomace (Figure 3). Depending on the apple variety used, the weight of pomace ranges between 20% and 30% of the dry matter of the fruit [30,31].

Apple pomace is a mixture of peel, core, seeds, calyx, stem, and soft tissue. It has a high water content of about 75% of the raw material and also contains insoluble carbohydrates such as cellulose, hemicellulose, and lignin. In addition, apple pomace contains sugars (glucose, fructose, and sucrose, which constitute 7%) as well as a large amount of minerals (magnesium, calcium, potassium, and phosphorus), pectin, proteins, and vitamins [32,33,34,36]. The chemical composition of apple pomace varies depending on the type of the processed raw material, the degree of maturity, the method used for pressing, and the number of pressing cycles [35]. Due to its rich composition (Table 1), pomace could be considered as a raw material for producing various products [36,37].

**Table 1:** Chemical composition of apple pomace (%)

Components	Kosmala et al. [38]	Skinner et al. [33]	Sato et al. [39]	Magyar et al. [40]
Dry matter	95.4	n.d.	88.57	n.d.
Protein	5.7	2.7–5.3	2.74	n.d.
Dietary fiber	61.1	4.4–47.3	43.63	40.3
Carbohydrates	25.3	44.5–57.4	39.35	20.2
Pectin	n.d.	3.2–13.3	n.d.	n.d.
pH	n.d.	n.d.	n.d.	n.d.
Ash	1.1	n.d.	1.80	2.2

n.d. – no data.

A number of scientific papers describing the bioconversion of pomace to various products, including phenols, lactic acid, vinegar, pectin, and ethanol, can be found in the available literature [37,41,42]. Currently, ethanol, the so-called first-generation biofuel, is produced in the USA using starch from maize grains [43,44]. However, the production of ethanol and other compounds from grains such as corn, wheat, sorghum, barley, and other edible ingredients is controversial. It is even considered as wastage of potential food [44,45]. One alternative is to utilize useless industrial waste, such as apple pomace, in the production of biofuel (the so-called second-generation biofuel) or other commercially significant compounds.

Apple pomace is produced all over the world, and its biodegradability is a serious environmental problem. It is estimated that the USA processes about 2.6 million Mg of apples into juices, producing about 1 million Mg of apple pomace per year [40]. Brazil produces approximately 800,000 Mg of apple pomace per year, which is mainly used as animal feed [46]. However, such use of apple pomace is limited due to its low protein and vitamin content, which implies that its nutritional value is low. In India, about 1 million Mg of apple pomace is produced annually from fruit pressing, but only about 10,000 Mg is further managed [47]. A study estimated that around 80 million Mg of apple pomace was produced worldwide in 2013 [48].

A problem associated with the management of valuable waste is unfavorable environmental changes. In the case of apple pomace, the major problems encountered are, *inter alia*, a high COD of 250–300 g/kg and spontaneous fermentation processes. Material and economic losses related to the storage of pomace in landfills are also an important issue. In the USA, the storage fees for apple pomace exceed $10 million per year. From the viewpoint of environmental protection, among plant wastes only hard seeds of certain fruits, for example, plums, cherries, apricots, or wild cherries, could be stored. Due to its high water content (up to 75%), apple pomace is quickly perishable and sensitive to high humidity, and the presence of biologically active compounds may lead to rapid microbiological contamination [49]. Hence, the large mass of pomace produced in a short period imposes a heavy burden on processing companies all over the world, and the logistics of this raw material seems to be one of the biggest challenges of its management.

## 3 Pomace application: current state and prospects

Most by-products produced during fruit and vegetable processing (e.g., apple processing) are considered as waste, most of which is disposed of at landfill sites or used as animal feed. However, the use of this waste as animal feed is limited due to rapid spoilage, microbial growth, and low nutritional value resulting from low levels of proteins and vitamins [50]. Currently, apple pomace is used in the production of fruit teas (dried pomace) [37,51] and pectin and fiber preparations [41,52]. The main aim of waste management is the biotransformation of the maximum possible amount of raw materials into useful, industrially valuable products through biotechnological processes involving various groups of microorganisms.

Apple pomace is a valuable resource, but its management is a major challenge for industries. Due to the presence of sugars, such as glucose, fructose, and sucrose, apple pomace has been studied for its potential use in the production of aromatic compounds [52,53], ethanol [35], organic acids [41], enzymes [54,55], protein-enriched feed [56,57,58], and edible fungi [59].

A study conducted by Madrera et al. [60] showed that fermentation of apple pomace with the use of yeast strains such as *Saccharomyces cerevisiae* and *Hanseniaspora* (*Hanseniaspora valbyensis* and *Hanseniaspora uvarum*) resulted in the production of about 132 volatile compounds. The quantity and quality of the aromatic compounds produced were strongly dependent on the yeast strains used. The study concluded that fermentation of apple pomace with the use of appropriate yeast strains may be an effective way to obtain aromatic compounds. This emphasizes that postfermentation apple pomace or extract could be widely used in the food industry as a natural fragrance, which will also help to increase the acceptance of products by the consumers, who show great interest in natural foods that have no additives of chemical origin.

Gulhane et al. [61] used apple pomace as a substrate for ethanol production. The following microorganisms were used in the production process: *S. cerevisiae*, *Fusarium oxysporum*, and *Aspergillus foetidus*. According to the results determined on the basis of iodometric titration, the highest ethanol content (1.37 g/100 g) was produced by a combination of all three tested fungi, the second highest was produced with fermentation using only *S. cerevisiae* (1.32 g/100 g), and the lowest was produced by a combination of *A. foetidus* and *F. oxysporum* (1.29 g/100 g). In another study, Magyar et al. [40] reported a yield of 134 g of ethanol/kg of pomace using *S. cerevisiae* ATCC 4124 yeast. These results show that apple pomace can serve as an excellent raw material for ethanol production. The waste from the agro-food industry, especially the fruit-processing industry, is ideal for the production of spirit, as the average amount of fermentable sugars contained in such waste is about 7%, which suggests that it is possible to obtain up to 4.4 L of pure ethyl alcohol from 100 kg of pomace.

Shojaosadati and Babaeipour [62] tested the use of apple pomace in the production of citric acid using *A. niger*. In the bioreactor culture, 124 g of citric acid was obtained from 1 kg of pomace with an efficiency of 80% per total sugar. Apart from the production of citric acid, the possibility of using pomace in the biosynthesis of lactic acid has also been verified. In a study by Gullón et al. [63], about 30 g/L of lactic acid was obtained after 6 h of fermentation with the use of *Lactobacillus rhamnosus* CECT-288 bacteria.

In a study carried out by Hang and Woodams [64], apple pomace was used as a substrate for the production of β-fructofuranosidase by mold strains of *Aspergillus fumigatus*, *A. foetidus*, and *A. niger*. It was found that *A. foetidus* produced the highest amount of the enzyme, with more than 900 U of β-glucosidase/kg of apple pomace, while *A. fumigatus* and *A. niger* produced 48 and 73 U, respectively. In a study performed by Dhillon et al. [65], apple pomace was tested as a raw material for cellulase production using *Aspergillus niger* NRRL-567. Another study by Zhong-Tao et al. [66] showed that after the fermentation of this waste product in solid form for 48 h at 30°C with the use of various mold strains from *A. niger* species, the amounts of pectinase, proteinase, and cellulase produced were 21,168, 3,585, and 1,208 U/g, respectively.

Pachapur et al. [48] evaluated the potential of apple pomace hydrolyzate (APH) and crude glycerin (CG) in hydrogen production using *Clostridium butyricum* and *Enterobacter aerogenes*. When used as the only carbon source, CG produced 19.46 mmol H_2_/L through glycerol metabolism reduction pathway, whereas APH favored the oxidative pathway, which resulted in a higher production of H_2_ (26.07 ± 1.57 mmol/L) and a lower production of by-products (1,3-propanediol and ethanol). Preliminary tests carried out by Olech et al. [67] showed that by-products of the agro-food industry, such as apple pomace, may also serve as substrates for biogas plants and can be used as alternatives to other substrates. Research has also been conducted on increasing the nutritional value of apple pomace to use it as animal feed. A study showed that fermentation of solid pomace using *C. utilis* and *Kloeckera apiculata* increased the protein content in pomace from 3% to 7% of the dry matter. Some studies were also performed to enrich apple pomace with *S. cerevisiae* [68,69].

Apple pomace is an edible fruit residue and can therefore be safely transformed into products that are useful to both humans and animals. The large amount of pomace produced annually and its rich composition suggest that, from an economic point of view, its use could be economically viable; for example, pomace could be used in the production of metabolites of various microorganisms that are widely used in industries. For several decades, both in industries and in the scientific world, a trend related to the efficient use of natural resources has been observed. Instead of disposing the agro-industrial residues in an unfavorable form in the environment, they can be bioconverted to various compounds with added commercial value using the biomass of microorganisms [70,71]. The above-cited examples show that microorganisms have a significant value in the processing of apple pomace.

## 4 Waste fat materials

Frying fat, or soap stock, is the main by-product produced during the refining of vegetable oils and is the major fat waste from the agro-food industry. It is most often used in the production of biosurfactants by yeasts of the *Candida* sp. Sophorolipids are an example of such extracellular surfactants. These compounds consist of a hydrophobic part, containing long-chain fatty acids connected by a β-glycosidic bond, and a hydrophilic part called sophorose. They can be used to reduce the surface tension of water, inhibit the action of free radicals in cosmetic applications, improve wound-healing processes, and stimulate the metabolism of skin fibroblasts [72,73]. Some species can produce biosurfactants when waste ingredients obtained from the food industry are added; one such species is *Candida bombicola*. In addition to biosurfactants, raw materials rich in fats can be used to obtain enzymes and biomass. In a study by Saenge et al. [74], the growth and fat-storing capacity of *Rhodotorula glutinis* were studied. The basic carbon source used for yeast cultivation was wastewater obtained during palm oil production. Due to the use of the *R. glutinis* TISTR 5159 strain, the COD of wastewater was reduced by 70%. In addition, the strain reduced the weight of waste to 9.2 g/L and lipid content by 60.6% (5.5 g/L) and produced 188.3 mg/L of carotene. Another study [75] reported the possibilities of producing biomass rich in microbiological fat using frying oils. *Y. lipolytica* was used for this purpose. Fats used in the media were by-products obtained by frying vegetables, fish, and chicken. Thus, these studies show a very high biotechnological potential of *Y. lipolytica* in waste management, implying that yeast can be used as a feed additive with the help of applied culturing technique.

## 5 Other postproduction wastes

Wastewaters from various sources including slaughterhouses, dairy industries, and compressor stations are rich in fatty raw materials. In particular, the management of wastewater produced during olive oil extraction is a challenge for Mediterranean countries. During phase separation using water steam, a part of the fatty phase is washed away with water and accumulates as wastewater rich in oily substances. The dairy industry also produces wastewater containing fats and hydrolyzates as well as long-chain fatty acids.

Recently, the concept of using production process residues as substrates for enzyme synthesis by yeast has been introduced. Until now, waste from the fat-processing industry has been successfully used for targeted enzyme production. Wastewater resulting from olive oil production has been effectively managed. The following yeast strains were found to be capable of producing extracellular enzymes: *Y. lipolytica*, *Candida rugosa*, and *C. cylindracea* [76]. Of these, *C. cylindracea* showed the highest production potential during liquid culture in flasks. The CBS 7869 strain of this species produced the maximum amount of enzyme (2,200 U/L). Next, the possibility of the production of these enzymes on an industrial scale was determined using the same strain in a periodical culture. The high amount of enzyme produced (3,511 U/L) indicated the potential of utilizing wastewater resulting from olive oil extraction in the production of lipolytic enzymes [77]. An additional advantage noted was the multiplication of yeast biomass, which can be used for the production of feed or as yeast extract.

Lycopene, β-carotene, cryptoxanthin, and astaxanthin are produced on an industrial scale by chemical synthesis. Carotene is also produced on an industrial scale using *Dunaliella* sp. algae. These microorganisms have been found to produce carotenoid at a level of 3–5% of dry matter [78]. Research studies focusing on finding a cheaper method for the production of carotenoids using microorganisms are underway. Some species of yeasts that have the ability to produce carotenoids include those of *Rhodotorula*, *Sporobolomyces*, *Phaffia*, *Rhodosporidium*, and *Sporidiobolus.* Industrial wastes that can be used to synthesize carotenoids include molasses, whey filtrate, soybean extract, concentrated waste from the production of grape syrup, hydrolyzed waste from the production of flour from mung beans, and radish waste [78]. Among these raw materials, molasses was found to be the best with a high productivity of carotenoids (125 mg/L). Aeration during cultivation, pH of the medium, culture time, and type and concentration of carbon source are important factors affecting the production of carotenoids [79]. Apart from the culture conditions, selection of the strain plays an important role. Studies have shown that the production of carotenoids can be improved by using mutagenic yeast strains with promising results. For instance, in a study by Bhosale et al. [80], mutant *R. glutinis* strains produced 120 times more carotenoids than nongenetically modified ones (17%). The cultures were carried out for 36 h on YM agar medium. The authors also attempted to improve the synthesis of carotenes by the addition of bivalent metal cations. In the control culture, the amount of carotenoids did not exceed 33 mg/L, whereas in the medium enriched with cations of zinc, calcium, and iron, the amount of carotenoids synthesized was found to be 68.8, 67.0, and 66.4 mg/L, respectively [80]. In another study by the same authors, the influence of high-salinity waters on the growth and synthesis of carotenoids by a mutant *R. glutinis* 32 strain was examined. The study showed that abundantly available seawater can be used as a substitute for distilled water in the cultivation of the *R. glutinis* 32 yeast. In the study by Bhosale et al. [80], the amount of carotenes synthesized in water rich in sodium chloride (86 mg/L) was higher compared to distilled water (70 mg/L). The culture was carried out at a temperature of 28°C in a fermenter with air stream feeding (0.7 L/min). The medium used in the study had a pH of 6.0. In another study, a mixed culture of bacteria and yeast species including *R. glutinis* and *Kluyveromyces lactis* was used to increase carotene production. *Lactobacillus helveticus*, homofermentative lactic acid bacteria, and *K. lactis* yeasts produced the enzyme β-D-galactosidase, which hydrolyzed lactose to galactose and glucose. The resulting hexose was used as a source of growth by the carotene-producing yeast species *Rhodotorula rubra*. Under optimized culture conditions, the amount of β-carotene synthesized was 248 μg/g [78].

## 6 Conclusion

The perspective of using microorganisms to protect the environment and to obtain individual metabolic products is promising. Given the current state of knowledge and assessment possibilities, targeted microbial activity in different environments should be increasingly considered as a means of obtaining SCP. Evidence of further processing of waste coming from all over the world builds a strong foundation for the industrial production (i.a., feed yeast, microbial biomass, vitamins, enzymes). Overall, the management of waste materials, especially those of agro-food origin, with the use of microorganisms can help in implementing a number of actions aimed at protecting the environment. The widest possible use of waste to obtain microbial metabolites opens up new possibilities for their industrial use. There is no universal method for the disposal of all waste, and therefore the process of using a given waste is selected according to the properties of the substances and individual groups of microorganisms. There are many methods of waste disposal, which differ from each other in terms of end products, degree of advancement, and environmental impact.
